# Cationic Lipid-Formulated DNA Vaccine against Hepatitis B Virus: Immunogenicity of MIDGE-Th1 Vectors Encoding Small and Large Surface Antigen in Comparison to a Licensed Protein Vaccine

**DOI:** 10.1371/journal.pone.0101715

**Published:** 2014-07-03

**Authors:** Anne Endmann, Katharina Klünder, Kerstin Kapp, Oliver Riede, Detlef Oswald, Eduard G. Talman, Matthias Schroff, Christiane Kleuss, Marcel H. J. Ruiters, Christiane Juhls

**Affiliations:** 1 MOLOGEN AG, Berlin, Germany; 2 Synvolux Therapeutics B.V., Groningen, The Netherlands; 3 Department of Pathology and Medical Biology, University of Groningen, Groningen, The Netherlands; CRCL-INSERM, France

## Abstract

Currently marketed vaccines against hepatitis B virus (HBV) based on the small (S) hepatitis B surface antigen (HBsAg) fail to induce a protective immune response in about 10% of vaccinees. DNA vaccination and the inclusion of PreS1 and PreS2 domains of HBsAg have been reported to represent feasible strategies to improve the efficacy of HBV vaccines. Here, we evaluated the immunogenicity of SAINT-18-formulated MIDGE-Th1 vectors encoding the S or the large (L) protein of HBsAg in mice and pigs. In both animal models, vectors encoding the secretion-competent S protein induced stronger humoral responses than vectors encoding the L protein, which was shown to be retained mainly intracellularly despite the presence of a heterologous secretion signal. In pigs, SAINT-18-formulated MIDGE-Th1 vectors encoding the S protein elicited an immune response of the same magnitude as the licensed protein vaccine Engerix-B, with S protein-specific antibody levels significantly higher than those considered protective in humans, and lasting for at least six months after the third immunization. Thus, our results provide not only the proof of concept for the SAINT-18-formulated MIDGE-Th1 vector approach but also confirm that with a cationic-lipid formulation, a DNA vaccine at a relatively low dose can elicit an immune response similar to a human dose of an aluminum hydroxide-adjuvanted protein vaccine in large animals.

## Introduction

Hepatitis B is a potentially life-threatening liver disease caused by the hepatitis B virus (HBV). It is a major global health concern as an estimated 2 billion people have been infected with the virus. About 360 million people live with chronic HBV infections which can later develop into liver cirrhosis or liver cancer and about 600,000 people die every year from HBV-related disease [1]. HBV contains three envelope proteins encoded within a single open reading frame. Depending on the translation initiation sites, three proteins are produced: (1) the small (S) protein as the major constituent of the HBV envelope and secreted surface antigen (HBsAg) particles, (2) the middle (M) protein containing the PreS2 domain at the N-terminus of the S protein, and (3) the large (L) protein containing a further addition of the PreS1 domain at the N-terminus of the M protein [2]. In natural infection with HBV, the envelope proteins can be secreted as subviral HBsAg particles that contain high amounts of S protein, variable amounts of M protein and traces of L protein embedded in host cell-derived lipids [3].

Recombinant expression of the S protein in yeast yields HBsAg particles which are the basis of currently marketed vaccines against HBV [4]. A three-dose series of these vaccines administered over a period of 6 months is recommended for protection against infection, which is considered to be correlated to S protein-specific (anti-HBs) antibody levels. Though conventional vaccines induce protective antibody responses in >90% of healthy adult recipients, they fail in non-responders like elderly, smokers, chronically ill or immuno-compromised vaccinees [5]. Thus, improved vaccines are still desirable.

Research and development of next generation vaccines against HBV comprise the use of novel adjuvants for recombinant HBsAg [4], [6], [7], [8], DNA vaccines [9], [10] as well as additional or optimized antigens [11], [12], [13]. The so-called third-generation vaccines contain PreS1 and PreS2 domains of HBsAg that harbor a number of epitopes relevant for attachment and uptake of HBV into hepatocytes. Neutralizing antibodies against these epitopes extend the protective capacity of a vaccine [14], [15]. Consequently, third-generation vaccines exhibited enhanced immunogenicity also in non-responders to conventional vaccines [11], [12], [13]. However, due to the necessary glycosylation of PreS1 and PreS2 domains, they must be produced in mammalian cell cultures. Thus, extra costs for manufacturing in comparison to yeast-derived vaccines have impeded marketing and introduction into clinical practice. Here, the use of DNA vaccine technology holds inherent benefits.

We have previously developed DNA vectors with reduced size, the Minimalistic Immunogenically Defined Gene Expression (MIDGE) vectors [16]. MIDGE-Th1 vectors are linear double-stranded DNA molecules, which are closed with single-stranded hairpin loops at both ends and contain a peptide nuclear localization sequence covalently bound to one of the loops. They exclusively comprise the expression cassette. Immunization with MIDGE-Th1 vectors elicits strong humoral and cellular immune responses [17], [18]. When formulated with the cationic lipid SAINT-18 [19], MIDGE-Th1 DNA vaccines induce significantly increased antibody responses against the S protein of HBsAg in mice [20].

In our work presented here, we aimed to develop a novel, effective, SAINT-18-formulated DNA vaccine against HBV. To this end, we constructed MIDGE-Th1 vectors encoding either the S or the L protein of HBsAg and characterized their expression pattern *in vitro* and evaluated their immunogenicity in mice. To demonstrate prophylactic efficacy in a large animal model, we compared our SAINT-18-formulated MIDGE-Th1 vector approach to the protein vaccine Engerix-B in pigs.

## Materials and Methods

### Ethics statement

The animal studies were carried out in strict accordance with German animal welfare regulations and Good Laboratory Practice (GLP) regulations at LPT (Laboratory of Pharmacology and Toxicology, Hamburg, Germany) with prior approval of LPT’s institutional animal care and use commissary (mouse study: 27619; pig study: 28295) and of the relevant authorities (mouse study: Behörde für Gesundheit und Verbraucherschutz, V 11307-591-00.33; pig study: Ministerium für Landwirtschaft, Umwelt und ländliche Räume, V 312-72241.123-1).

### Construction and synthesis of MIDGE-Th1 vectors

**Figure 1:**
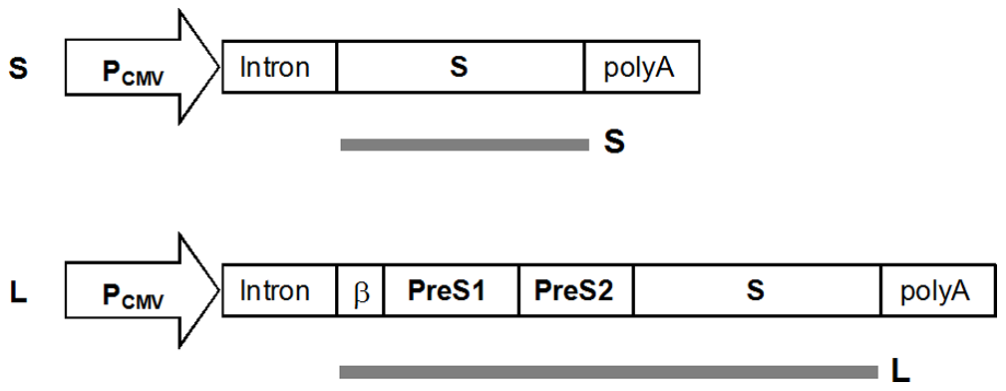
Schematic diagram of the expression vectors containing the S or L protein coding sequence from HBV. The expected protein products are indicated as thick gray lines below the respective coding sequences. β = β-lactamase secretion signal sequence.

The coding sequence of the L protein of HBsAg containing the PreS1, PreS2 and S coding sequences (subtype *adw2*, GenBank: AM282986.1 [21]) was optimized for human codon usage and synthesized by GeneArt (Regensburg, Germany). The first 7 amino acids of the LHBsAg coding sequence were replaced by the signal sequence of β-lactamase from plasmid pBluescript KS(+) (Stratagene, Santa Clara, USA) (5′-ATGAGTATTCAACATTTCCGTGTCGCCCTTATTCCCTTTTTTGCGGCAT TTTGCCTTCCTGTTTTTGCTCACCCAGAAACGCTGGTGAAGTAAAA) as previously described [22] to yield the antigen coding sequence for the L protein expression vector (Figure 1). The same S coding sequence as in the L protein expression vector was used as antigen coding sequence to construct the S protein expression vector (Figure 1). The synthesis of MIDGE-Th1 vectors has been described previously [17]. Briefly, the antigen coding sequences were inserted into plasmids containing *Eco*31I recognition sites flanking the expression cassette, which consists of the CMV immediate-early enhancer/promoter, a chimeric intron composed of the 5′-donor site from the first intron of the human β-globin gene, the branch point, and 3′-acceptor site from the intron of an immunoglobulin gene heavy chain variable region and a SV40 polyadenylation signal. The expression cassette was released by *Eco*31I (Thermo Scientific, Vilnius, Lithuania) digestion of the plasmids. Subsequently, each end of the cassette was protected against exonuclease digestion by ligating a specific hairpin oligodeoxyribonucleotide (ODN) with one of them crosslinked to a NLS peptide (PKKKRKVEDPYC). Unprotected DNA fragments were digested using the exonuclease activity of T7 DNA Polymerase (Thermo Scientific) and the MIDGE-Th1 products (MIDGE-S-Th1∶1,881 bp; MIDGE-L-Th1∶2,458 bp) were purified by anion exchange chromatography (Fractogel EMD DMAE, Merck KGaA, Darmstadt, Germany) and concentrated by precipitation with 96% (v/v) ethanol.

### *In vitro* expression of HBsAg

The expression of the S and L proteins from DNA vectors was examined in CHO-K1 cells (ATCC CCL-61). Cells were grown in Ham’s F-12 medium supplemented with 10% (v/v) inactivated fetal bovine serum (FBS) and 100 U/ml penicillin and 100 µg/ml streptomycin at 37°C in 5% CO_2_. Prior to transfection, cells were detached by trypsin and suspended in Ham’s F-12 without FBS and antibiotics. Then, 2–3×10^6^ cells were transfected with 20 µg DNA (if not indicated otherwise) by electroporation (single pulse, 270 V and 1,650 µF). As control, cells were transfected with eGFP-encoding vector.

24 h after transfection, cells were lysed by nitrogen decompression (Cell Disruption Vessel 4639, Parr Instruments, Moline, USA). Lysates were centrifuged at 1,000 × *g* for 1 min to sediment the nuclei. Supernatants were centrifuged at 14,000 × *g* for 30 min to sediment the membrane-containing fraction. The pellet was suspended in 60 µl of Hepes buffer (20 mM Hepes pH 8.0, 1 mM DTT, 1 mM EDTA pH 7.6) supplemented with protease inhibitors. The total protein amount was determined via Bradford assay. Protein (10 µg) was mixed with Laemmli buffer, then subjected to SDS-PAGE (12% (w/v) SDS-polyacrylamide gel) and blotted onto nitrocellulose membrane (Optitran BA-S85, Whatman, Piscataway, USA). Blocking was performed with Roti-Block (Roth, Karlsruhe, Germany). For detection of the S protein of HBsAg, S protein-specific horse serum (ab9193, Abcam, Cambridge, UK) at 1∶1,000 dilution and HRP-conjugated rabbit anti-horse IgG (ab6921, Abcam) at 1∶20,000 dilution were used. For detection of the PreS1 domain, PreS1-specific mouse serum (Proteogenix, Oberhausbergen, France) at 1∶1,000 dilution and HRP-conjugated goat anti-mouse IgG (A9917, Sigma-Aldrich, St. Louis, USA) at 1∶20,000 dilution were used. HRP activity was detected with SuperSignal West DURA Extended Duration Substrate (Fisher Scientific, Schwerte, Germany) and visualized by a CCD camera.

Secreted HBsAg was analyzed in cell culture supernatants 72 h after transfection by ELISA MonolisaTM HBsAg ULTRA (Bio-Rad, Redmond, USA) according to the manufacturer’s instructions and applying recombinant HBsAg (ProSpec-Tany Technogene, East Brunswick, USA) as quantification standard.

### Immunogenicity study in mice

Female BALB/c mice, 12 weeks of age at the first administration and weighing 17.8–21.4 g, were supplied by Charles River Laboratories (Sulzfeld, Germany). All mice were acclimatized for 7 days before immunization. Mice (n = 6 per group, allocated by means of a computer randomization program) were immunized intradermally (i.d.) into the dorsal skin of the tail base with formulations of 10 µg MIDGE-Th1 vectors (c = 2 mg/ml) with 1 µl of 7.5 mM SAINT-18 in water (at the optimal w/w ratio MIDGE-Th1/SAINT-18 of 1∶0.5 [23]) on test days 1 and 21. As negative control, mice received SAINT-18 only. On test day 35, blood was collected from the *retrobulbar venous plexus* under ether anaesthesia and animals were sacrificed by cutting the *aorta abdominalis*. Sera were obtained and HBsAg specific IgG1 and IgG2a antibodies determined as previously described [20]. The detection limit of the ELISA was 39 pg/ml for IgG1 and IgG2a.

For determination of PreS1-specific antibodies in sera of mice, ELISA plates were coated with 1 ng/µl PreS1 antigen (Abzymo biosciences, Beijing, China). As detection antibody, HRP-conjugated goat anti-mouse IgG (115-035-003, Jackson ImmunoResearch, West Grove, USA) at 1∶15,000 dilution was applied. HRP activity was detected using Substrate Reagent Pack (DY999, R&D Systems, Minneapolis, USA). The optical density (OD) was measured at 450 and 595 nm. The titers were determined as the highest serum dilution yielding an OD_450–595_ more than twice as high than the mean OD_450–595_ of the negative control group. Determination of PreS2-specific antibodies was performed as described for PreS1 except that ELISA plates were coated with 1 ng/µl of an equimass mixture of three peptides spanning the PreS2 amino acid sequence (amino acid 4–21, amino acid 22–38, amino acid 39–55; Proteogenix).

### Immunogenicity study in pigs

Male outbred Landrace pigs, 9–10 weeks of age at the first administration and weighing 16.1 kg – 22.9 kg were supplied by BHZP GmbH (Dahlenburg-Ellringen, Germany). All pigs were acclimatized for 2 weeks before immunization and allocated to six groups (Table 1) by means of a computer randomization program. Pigs were immunized i.d. into the dorsal area of the left and right ear (alternating between immunizations) with formulations of MIDGE-Th1 vectors with SAINT-18 at the optimal w/w ratio MIDGE-Th1/SAINT-18 of 1∶0.5 [23] (Table 1). For comparison, pigs received SAINT-18 only at the same injection volume as the high dose groups of the MIDGE-Th1/SAINT-18 vaccine candidates. As positive control, pigs were immunized intramuscularly (i.m.) into the left and right side (alternating between immunizations) of the neck with an adult-human dose of Engerix-B (GlaxoSmithKline Biologicals, Rixensart, Belgium). Pigs were primed on test day 1 and boosted on test days 29 and 57. After the last blood sampling, the animals were returned to LPT’s animal stock as a source of plasma or serum samples.

**Table 1:** Study design - immunization of pigs with MIDGE-Th1 DNA vaccine candidates and protein vaccine Engerix-B.

Test item	Group	DNA or protein dose (µg)	Injection volume (µl)	Route	No. of pigs
Negative Control (SAINT-18)	Ctrl.	0	200	i.d.	3
MIDGE-S-Th1/SAINT-18	low S	83	50	i.d.	5
MIDGE-S-Th1/SAINT-18	mid S	167	100	i.d.	4
MIDGE-S-Th1/SAINT-18	high S	333	200	i.d.	5
MIDGE-L-Th1/SAINT-18	high L	333	200	i.d.	5
Engerix-B (human dose)	Engerix-B	20	1000	i.m.	4

### Measurement of antibody responses in pigs

At various time points, blood was collected from the *vena cava superior* of each animal into serum separator tubes (Sarstedt, Nürnbrecht, Germany) and allowed to clot for approximately 0.5 h. Afterwards, the samples were centrifuged at 4,200 × *g* for 5 min at room temperature. Total S protein-specific antibodies were determined using the Enzygnost Anti-HBs II kit according to the manufacturer’s instructions.

The HBsAg-coated ELISA plates of the Enzygnost Anti-HBs II kit were used to measure S protein-specific IgG1 and IgG2 antibodies. Mouse anti-porcine IgG1 and mouse anti-porcine IgG2 (MA1-80545 and MA1-80546, Thermo Scientific, Rockford, USA), diluted 1∶250 and 1∶100, respectively, served as secondary antibodies, and HRP-conjugated goat anti-mouse IgG (115-135-146, Jackson ImmunoResearch), diluted 1∶30,000, was used for detection. The level of S protein-specific IgG1 and IgG2 was determined as RLU/ml based on a standard derived from pooled sera of animals positive for total S protein-specific antibodies (measured as described above). The response was set to 8192 RLU/ml. Calibration standards were prepared by diluting the pooled positive porcine serum. The lowest calibration standard (16 RLU/ml for IgG1; 8 RLU/ml for IgG2) was accepted as lower limit of quantification.

PreS1-specific and PreS2-specific antibodies were determined as described in 2.4, except that HRP-conjugated goat anti-pig IgG (ab6915, Abcam) at 1∶15,000 dilution served as detection antibody. For every animal, the endpoint dilution titer was expressed as the highest reciprocal dilution of test serum yielding an OD_450–595_ more than twice as high than that obtained with the same dilution of pre-immune serum. For graphical and statistical purposes, samples tested negative were set to a titer of 10.

### Measurement of cellular responses in pigs

On test days 43 and 71, blood was withdrawn from the *vena cava superior* and collected into lithium-heparin-collection tubes (Sarstedt). The samples were subjected to a density centrifugation using Histopaque (Sigma-Aldrich).

The obtained peripheral blood mononuclear cells (PBMC) were suspended in medium (RPMI 1640, Invitrogen, Carlsbad, USA) supplemented with 10% (v/v) FBS, 100 U/ml penicillin and 100 µg/ml streptomycin and 2 mM α-glutamine. The cells were activated *in vitro* by addition of peptide pools (2 µg/ml for each peptide). Peptide pools consisted of 15-mer peptides with 11-mer overlaps covering the S, PreS1 and PreS2 amino acid sequence (peptides&elephants, Potsdam, Germany). As positive and negative control, PBMC were incubated with ConA (10 µg/ml) and with medium only. The ELISpot was carried out in duplicates using Porcine IFN-γ ELISpot Kit (EL985, R&D) with an incubation time of 18 hours. The ELISpot analysis was conducted using ImmunoScan Entry (CTL Europe, Bonn, Germany). The number of IFN-γ-secreting cells per 1×10^6^ PBMC was expressed as the difference between the number of spots in the stimulated wells and the number of spots in the medium control wells.

### Statistical analysis

Data were analysed with SPSS 15.0 (SPSS Inc., Chicago, USA). Normal distribution of data was confirmed using Kolmogorov-Smirnoff test prior to analyzing differences between the means of several experimental groups using one-way ANOVA and subsequent post-hoc analysis (two-sided Dunett’s test for multiple comparisons of treatment groups with negative control group; Tukey’s test for multiple pairwise comparisons of treatment groups). The significance level α was set to 5%.

## Results

### Construction of MIDGE-Th1 vectors and expression of HBV antigens *in vitro*

Conventional Hepatitis B vaccines are based on the S protein of HBsAg, so for direct comparison we generated an expression vector encoding the same antigen (S in Figure 1). Aiming to develop a hepatitis B vaccine inducing a broader set of antibodies than conventional protein vaccines and thereby increasing efficacy in weak or non-responders, we encoded the L protein in our second vector and thus included the DNA sequences of the PreS1 and PreS2 domains. Keeping the viral amino acid sequences, the DNA sequences of the S and the L protein were codon-optimized for enhanced expression in humans. Internal transcriptional and translational start sites were removed from the L protein coding sequence to express predominantly the full-length L protein, and the β-lactamase signal sequence was added to mediate secretion [22] (L in Figure 1). The optimized synthetic antigen coding sequences were each placed under control of a strong viral P_CMV_ promoter.

**Figure 2:**
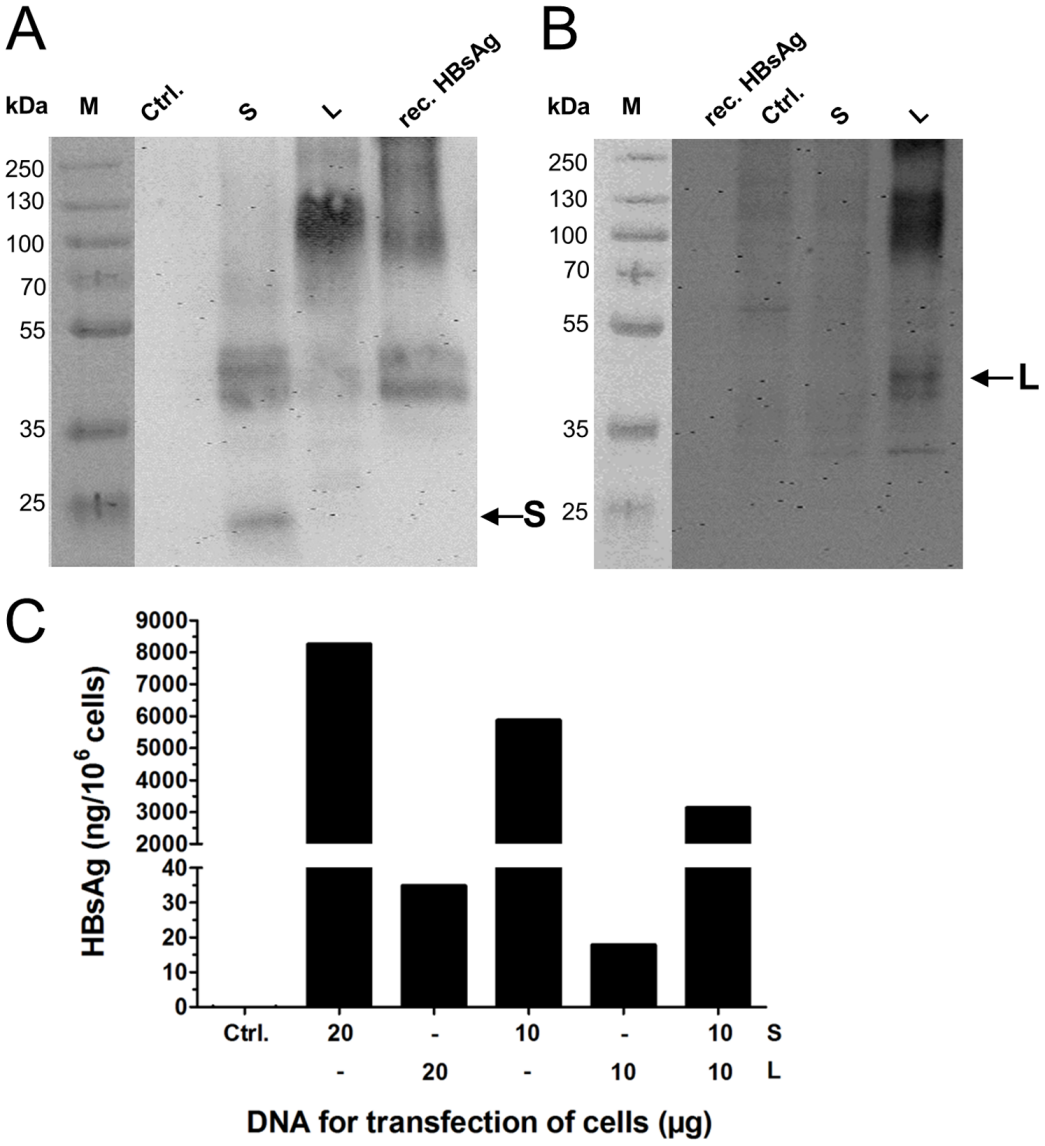
Expression of HBV antigens in CHO-K1 cells. Cells were transfected with expression vectors encoding the S or the L protein or eGFP as control (Ctrl.). Expression in cell lysates was detected by Western Blot using polyclonal S protein-specific antibody (A) or PreS1-specific mouse serum (B). rec. HBsAg: recombinant S protein of HBsAg. (C) Cells were transfected with the indicated amounts of single expression vectors or mixtures thereof. S protein expression was analyzed by ELISA in supernatants of cells 72 h after transfection.

Subsequently, CHO-K1 cells were transfected with the vectors and HBsAg expression was confirmed by Western blot analysis with an S protein-specific primary antibody (Figure 2A). In cells transfected with the S protein-encoding vector, the non-glycosylated form of the S protein was detected (molecular weight: ∼24 kDa [3]). Furthermore, dimers of the S protein at higher molecular weight were visible that were also detected in the positive control (recombinant S protein of HBsAg). In cells transfected with the L protein-encoding vector, the L protein was detected (bands between 35 and 55 kDa for monomers and between 100 and 130 kDa for multimers) due to its S protein domain. Expression of the full length L protein was confirmed with polyclonal mouse serum specific for the PreS1 domain (Figure 2B). Cells transfected with the L protein-encoding vector expressed the non-glycosylated and glycosylated forms of the L protein (molecular weight: 39 and 42 kDa [3]). Additionally, multimers of the L protein were detected at higher molecular weight. A lower molecular weight band of ∼32 kDa was probably due to unspecific binding of the polyclonal mouse serum.

Next, we evaluated secretion of antigens into the supernatant of transfected cells. We hypothesized that a mixture of MIDGE-Th1 vectors encoding S and L protein was a possible scenario for a novel HBV vaccine to induce strong anti-S antibodies and strong anti-PreS1 and anti-PreS2 antibodies. Thus, CHO-K1 cells were transfected with individual vectors or with an equimass mixture and supernatants were analyzed for the presence of secreted surface antigens by S protein-specific ELISA. Surprisingly, cells transfected with S protein-encoding vector secreted ∼250–300-fold more HBsAg than cells transfected with the same DNA amount of L protein-encoding vector (Figure 2C). Cells transfected with a mixture of each 10 µg S and 10 µg L protein-encoding vector secreted ∼50% less HBsAg in comparison to cells transfected with 10 µg S protein-encoding vector. Obviously, co-expression of S and L protein impaired HBsAg secretion.

### Immunogenicity study in mice

**Figure 3:**
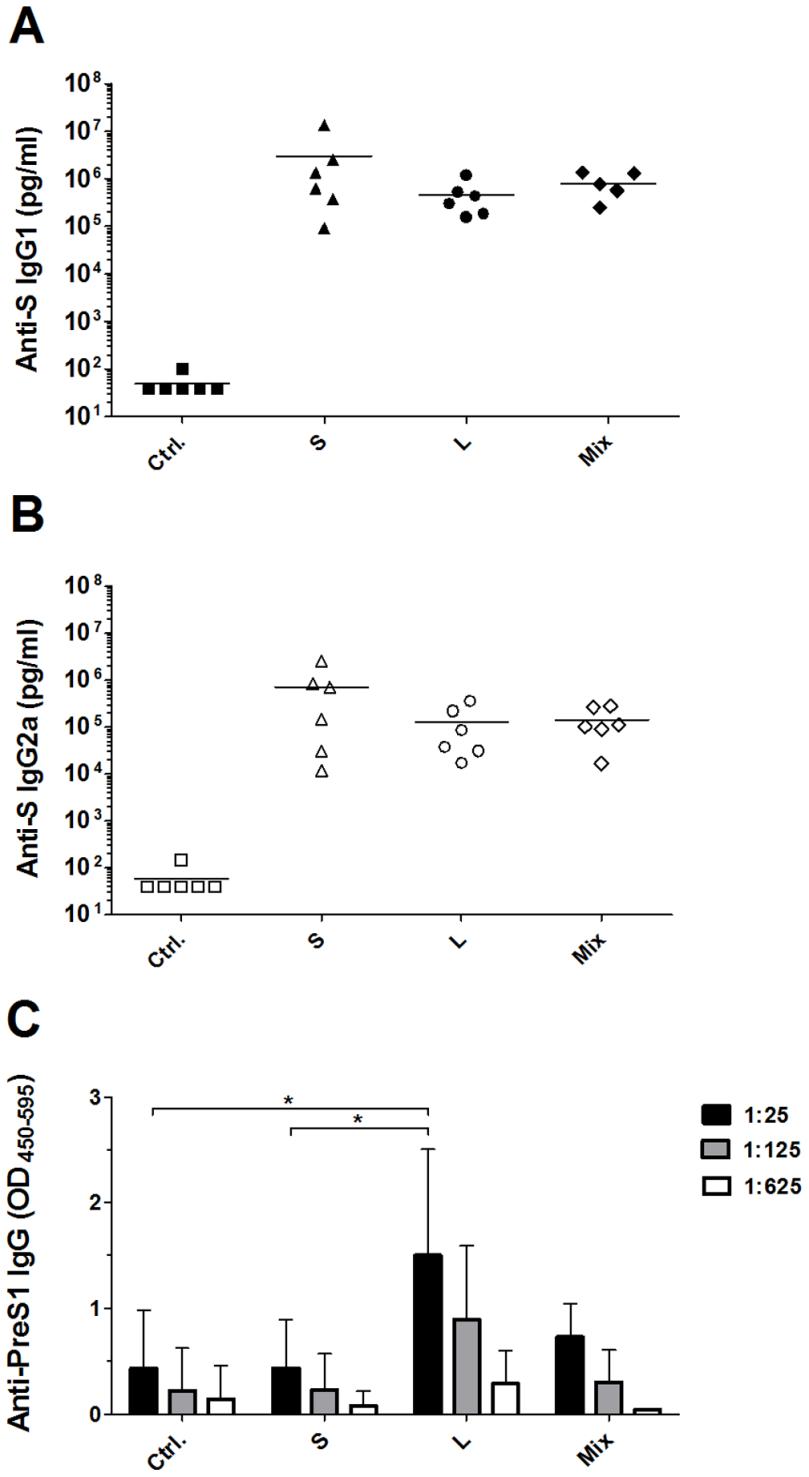
HBV antigen-specific humoral immune responses in mice after two intradermal immunizations with SAINT-18 (Ctrl.), 10 µg MIDGE-Th1 vectors encoding the S or the L protein or an equimass mixture of these vectors (Mix). MIDGE-Th1 vectors were formulated with SAINT-18. Mice were immunized on day 1 and 21 and serum samples were analyzed by ELISA for S protein-specific IgG1 (A) and IgG2a (B) and for PreS1-specific IgG (C) on day 35. (A), (B): Each symbol represents an individual mouse serum sample. The mean is shown for each group as horizontal line (n = 6). (C): PreS1-specific IgG were measured at 1∶25, 1∶125 and 1∶625 dilution and OD_450–595_ values expressed as mean and SD of six mice per group. Statistical analysis: (A, B) ANOVA, not significant. (C) Dunnett, L/Ctrl. 1∶25 dilution: *p = 0.028; Tukey, L/S 1∶25 dilution: *p = 0.043; other dilutions and group comparisons were not significant.

We next explored how the level of *in vitro* protein expression and secretion translates into the generation of anti-S humoral immune responses *in vivo* and whether PreS1- and PreS2-specific antibodies are induced. BALB/c mice were immunized with 10 µg MIDGE-Th1 vectors encoding either the S or the L protein or with 10 µg of an equimass mixture of these vectors on days 1 and 21. The DNA was formulated with SAINT-18 and administered i.d. to induce strong immune responses [20]. The concentration of S protein-specific antibodies was determined by ELISA in sera collected 14 days post boost immunization. S protein-encoding vectors elicited 6-fold and 4-fold higher S-specific IgG1 levels than L protein-encoding vectors and the equimass mixture, respectively (Figure 3A). S-specific IgG2a levels were also 6-fold and 5-fold higher in mice immunized with S protein-encoding vectors compared to L protein-encoding vectors and the vector mixture, respectively (Figure 3B).

Mice vaccinated with MIDGE-L-Th1 developed a significant anti-PreS1 immune response (Figure 3C), albeit with a rather low antibody titer (1∶125). The vector mixture induced no measurable anti-PreS1 response (Figure 3C). PreS2-specific antibodies were not detected in any group (data not shown). In summary, we did not observe a benefit of immunization with the vector mixture over the single MIDGE-Th1 vectors encoding either the S or the L protein in mice. Therefore, we proceeded with the single vectors and examined immunogenicity and efficacy of these vaccine candidates in a large animal model.

### Immune responses of pigs to S and L protein of HBsAg

**Figure 4:**
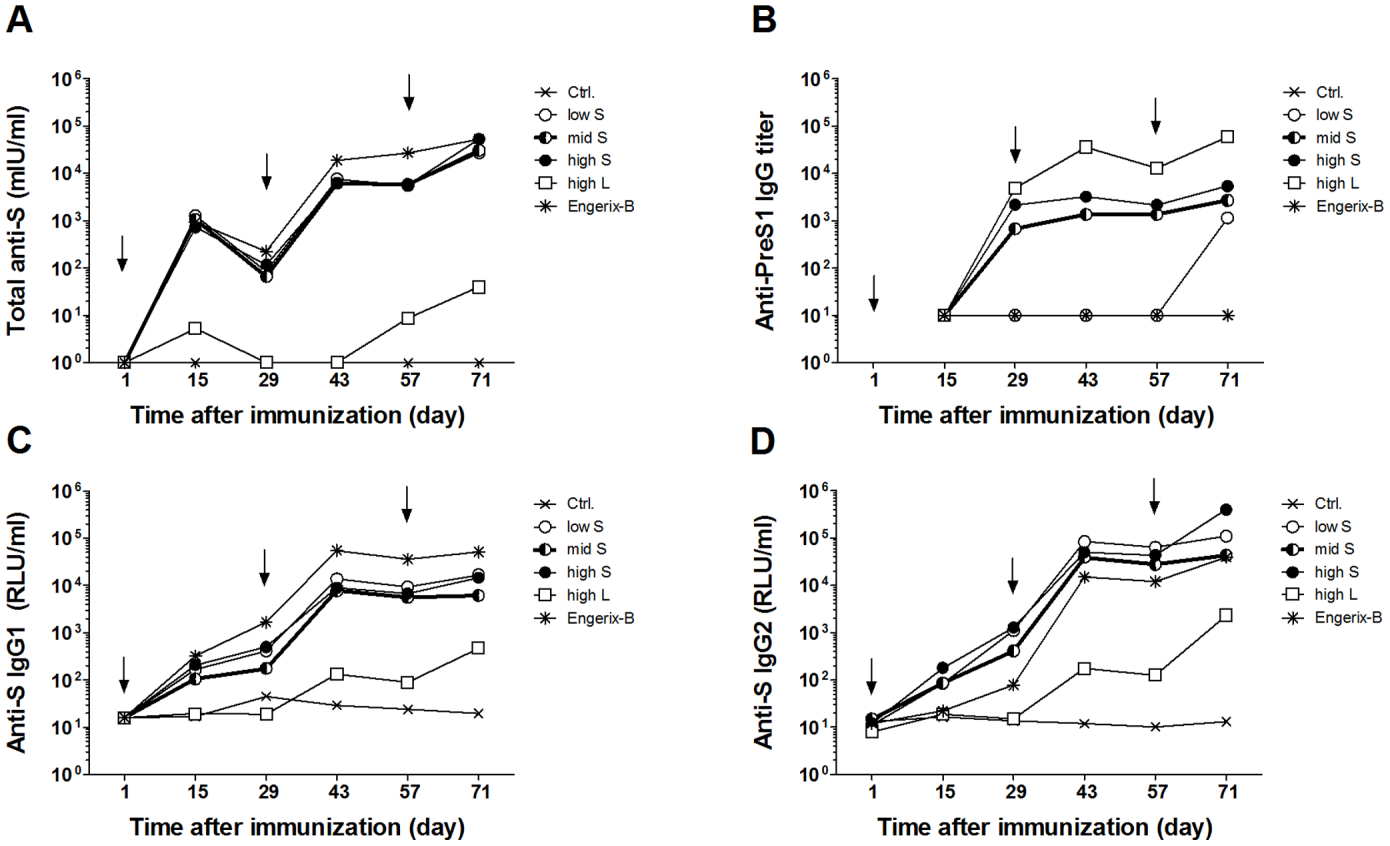
Kinetics of HBV antigen-specific humoral immune responses in pigs. Pigs were immunized with SAINT-18 (Ctrl.), a low, mid or high dose of SAINT-18-formulated MIDGE-Th1 vectors encoding the S protein (low S, mid S, high S), a high dose of SAINT-18-formulated MIDGE-Th1 vectors encoding the L protein (high L) or a human dose of Engerix-B on days 1, 29 and 57 (↓). Serum samples were collected on various test days and analyzed by ELISA for total S protein-specific antibodies (A), PreS1-specific IgG (B), S protein-specific IgG1 (C) and IgG2 (D). Data points represent mean values for all animals in each group (n = 3–5). For graphical purposes, samples without a PreS1-titer were set to 10 in (B). Statistical analysis is presented in Tables S1–S4.

**Figure 5:**
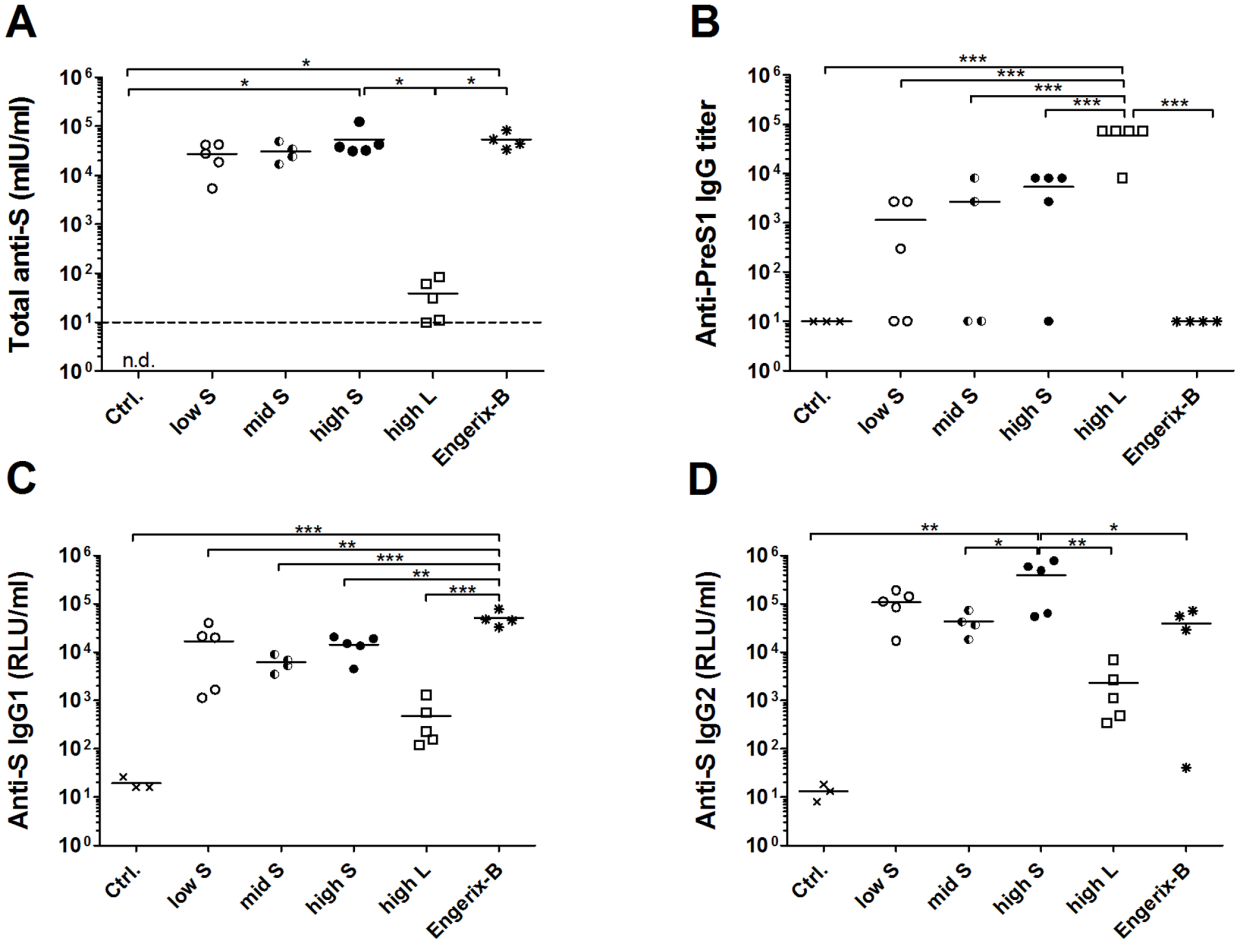
HBV antigen-specific humoral immune responses in pigs after three immunizations. Pigs were immunized with MIDGE-Th1 DNA vaccine candidates and Engerix-B on days 1, 29 and 57 (see legend Figure 4) and serum samples were analyzed by ELISA for total S protein-specific antibodies (A), PreS1-specific IgG (B), S protein-specific IgG1 (C) and IgG2 (D) on day 71. Each symbol represents an individual pig serum sample. The mean is shown for each group as horizontal line (n = 3–5). For graphical and statistical purposes, samples without a titer were set to 10 in graph (B). Statistical analysis: (A) Dunnett, high S/Ctrl. *p = 0.011, Engerix-B/Ctrl. *p = 0.015; Tukey, Engerix-B/high L *p = 0.019, high S/high L *p = 0.012. (B) Dunnett, high L/Ctrl. ***p = 0.00002, Tukey, high L/low S ***p = 0.00002, high L/mid S ***p = 0.00007, high L/high S ***p = 0.00006, high L/Engerix-B ***p = 0.00004 (C) Dunnett, Engerix-B/Ctrl. ***p = 0.00002; Tukey, Engerix-B/low S **p = 0.002, Engerix-B/mid S ***p = 0.0002, Engerix-B/high S **p = 0.001. Engerix-B/high L ***p = 0.00003. (D) Dunnett, high S/Ctrl. **p = 0.006; Tukey, Engerix-B/high S *p = 0.024, high L/high S **p = 0.007, mid S/high S day 71: *p = 0.026. Other group comparisons were not significant.

To establish the proof of concept of the SAINT-18-formulated MIDGE-Th1 DNA vaccine candidates, we compared their immunogenicity and protective efficacy with a full human dose of the licensed, aluminium-hydroxide-adjuvanted protein vaccine Engerix-B in pigs. The total S protein-specific antibody level in serum is an established surrogate parameter for protection of prophylactic vaccines against HBV and a level of 10 mIU/ml is considered to be protective against HBV infection in humans [5]. We immunized 9–10 weeks old male pigs with SAINT-18-formulated MIDGE-Th1 vectors or Engerix-B on days 1, 29 and 57 (Table 1). MIDGE-Th1 vectors encoding the S protein were tested in a low, intermediate (mid) and high dose to examine the influence of the dose on the strength of immune response. In consequence of its lower immunogenicity in mice, pigs received only the highest possible dose of the L protein-encoding vector. At various time points blood samples were collected and anti-S (total Ig, IgG1 and IgG2), anti-PreS1 (IgG) and anti-PreS2 (IgG) antibodies determined in sera. Figure 4 shows the kinetics of the humoral immune response and Figure 5 presents individual animal data after three immunizations on day 71. The results of the statistical data analysis are listed in Tables S1–S4.

Considerable levels of total S protein-specific antibodies were detected in the three MIDGE-S-Th1/SAINT-18 dose groups (Figures 4A and 5A). Indeed, all animals seroconverted (levels ≥10 mIU/ml) already 14 days after a single immunization (day 15, individual animal data not shown). With every injection, the antibody response was boosted. The strongest response was observed at the highest dose level two weeks after the third immunization (day 71). At any time point, differences among the MIDGE-S-Th1/SAINT-18 dose groups were not significant (Table S1).

Engerix-B elicited a similar level of total S protein-specific antibodies as MIDGE-Th1 vectors encoding the S protein. Differences were only significant for the low and mid dose on day 29 (Table S1) and disappeared on day 71 (Figure 5A). Comparing the S protein-specific IgG1 and IgG2 response, a striking difference between immunization with S protein-encoding MIDGE-Th1 vectors and Engerix-B became evident (Figure 4C and D; Figure 5C and D): Engerix-B induced approximately three- to eight-fold more IgG1 and 10-fold less IgG2 than MIDGE-S-Th1/SAINT-18. These differences were significant at various test days (Tables S3 and S4).

L protein-encoding vectors induced 1,000-fold lower levels of total S protein-specific antibodies than S protein-encoding vectors (Figure 4A) but still, four out of five animals responded (levels ≥10 mIU/ml) two weeks after the third immunization (Figure 5A). Furthermore, this vaccine candidate induced significant anti-PreS1 titers in comparison to the negative control group (Figure 4B, Figure 5B, Table S2). Three out of five animals were already positive at day 29 (28 days after a single immunization, individual animal data not shown). The titers increased with each subsequent immunization (Figure 4B) and all animals were positive two weeks after the third immunization (Figure 5B). Unexpectedly, some animals immunized with the S protein-encoding vector also developed anti-PreS1 titers. PreS2-specific antibodies were not detected in any treatment group (data not shown).

**Figure 6:**
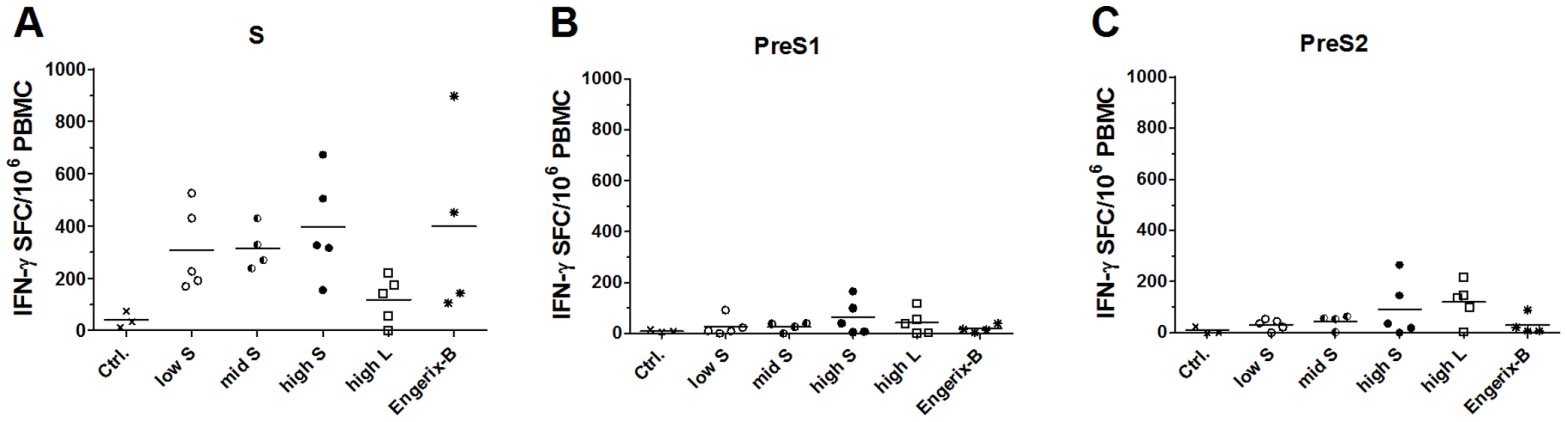
HBV antigen-specific cellular immune responses in pigs after three immunizations. Pigs were immunized with MIDGE-Th1 DNA vaccine candidates and Engerix-B (see legend Figure 4) and PBMC were obtained from blood collected on day 71. The cells were activated *in vitro* by addition of S protein peptide pool (A), PreS1 peptide pool (B) or PreS2 peptide pool (C). The number of IFN-γ-spot forming cells (SFC) per 10^6^ PBMC was expressed as the difference between the number of spots in the stimulated wells and the number of spots in the medium control wells. Each data point represents an individual sample and group means are indicated as horizontal lines. Statistical analysis: (A, B, C) ANOVA, not significant.

To assess cellular immune responses, PBMC were analyzed for antigen-specific IFN-γ-secreting cells by ELISPOT two weeks after the second (data not shown) and third immunization (Figure 6). Pigs immunized with the S protein-encoding vector at different doses developed clearly more S protein-specific IFN-γ-secreting PBMC than pigs of the negative control group (Figure 6A). Here we found a slight, albeit non-significant, effect of the DNA dose. The highest dose of MIDGE-S-Th1/SAINT-18 induced the same level of IFN-γ-secreting PBMC as Engerix-B whereas MIDGE-L-Th1/SAINT-18 induced only few S-specific IFN-γ-secreting PBMC.

Upon stimulation with PreS1 and PreS2 peptide pools, we observed only very low numbers of IFN-γ-secreting cells (Figure 6B and C). Pigs immunized with L protein-encoding vectors exhibited more IFN-γ-secreting PBMC than pigs of the negative control group. However, comparable numbers of IFN-γ-secreting PBMC were detected in animals immunized with S protein-encoding vectors at the same dose. Thus, the detected cellular responses were not considered to be specific for PreS1 and PreS2.

### Duration of humoral immune response in pigs

To assess whether formulated MIDGE-Th1 DNA vaccines conferred long-lasting antibody responses, we monitored two animals each per treatment group low and high dose MIDGE-S-Th1/SAINT-18 and Engerix-B for 6 months after the third immunization. Animals were selected for further monitoring based on total S protein-specific antibodies on day 71 (Table S5).

**Figure 7:**
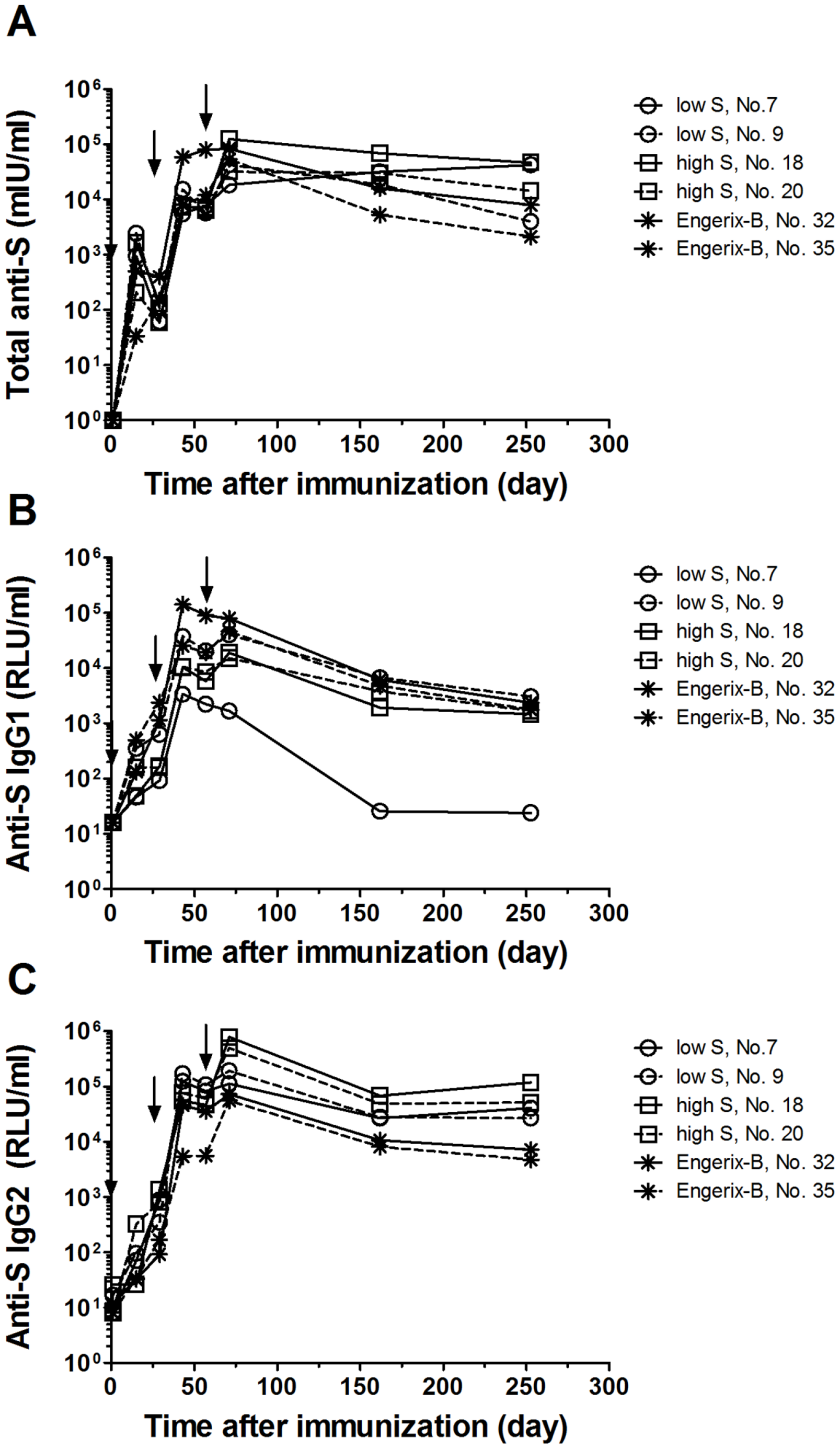
Duration of HBV antigen-specific humoral immune responses in individual animals. Pigs were immunized with MIDGE-Th1 DNA vaccine candidates and Engerix-B (see legend Figure 4) and serum samples were collected and analyzed by ELISA for total S protein-specific antibodies (A), S protein-specific IgG1 (C) and IgG2 (C) on various test days up to 6 months after the third immunization. The animal number is shown in the legend of the graph.

From day 71 to day 253, a ∼2.5-fold reduction of total anti-S antibody levels was observed in animals (No. 18 and 20) immunized with S protein-encoding vectors at high dose. The contraction was more pronounced in Engerix-B-immunized animals with 10-fold (animal 32) respectively 24-fold (animal 35) reduced levels (Figure 7A). Animal 7 of the low dose MIDGE-S-Th1/SAINT-18 group showed a 2-fold increase of total anti-S antibody levels, whereas the other animal (No. 9) exhibited a 10-fold reduction in the same period. All six animals still had protective antibody levels at the end of the study regardless of the vaccine they had received.

The S protein-specific IgG1 levels decreased ∼10-fold in animals immunized with S protein-encoding vectors from day 71 to day 253, except for animal 7 for which levels had dropped to background already by day 162 (Figure 7B). Again, levels in animals immunized with Engerix-B decreased strongly (∼25-fold from day 71 to day 253).

From day 71 to day 162 the S protein-specific IgG2 levels decreased ∼7–10-fold in all animals. Thereafter, S protein-specific IgG2 levels of animals immunized with S protein-encoding vectors remained constant until day 253, whereas the levels in animals immunized with Engerix-B further decreased over time (Figure 7C).

## Discussion

In the work reported here, we aimed to develop an improved hepatitis B vaccine and for this purpose, used SAINT-18-formulated MIDGE-Th1 vectors to express the S or the L protein of HBsAg. HBV DNA vaccines generally induce stronger humoral immune responses when epitopes are presented on secreted subviral particles [24]. The expected strong secretion of the S protein was confirmed in CHO-K1 cells transfected with the S protein-encoding vector. L protein secretion was intended to be enhanced by insertion of the β-lactamase secretion signal sequence as previously reported by others [22], [24], but our cell culture assays showed that the L protein was, nevertheless, mainly retained intracellularly. Co-transfection of CHO-K1 cells with S and L protein-encoding vectors hampered HBsAg secretion.

Surprisingly, the intracellular retention of the L protein was not reflected in a correspondingly low immunogenicity of the L protein-encoding vector in mice. In fact, the difference in S-specific antibody responses induced by S and L protein-encoding MIDGE-Th1 vectors was less pronounced than anticipated from the ∼250–300-fold difference in antigen secretion observed *in vitro*. This suggests that low amounts of secreted antigen are sufficient to induce antibodies in mice and that excess amounts of S protein expressed from MIDGE-S-Th1 do not necessarily further increase antibody levels. It is also conceivable that intracellularly retained L protein contributed to the induction of humoral immune responses as shown before for DNA immunization with other L protein expression vectors [25], [26]. In contrast to a distinctive humoral immune response to PreS1, no PreS2-specific antibodies were detected after immunization of mice with the L protein-encoding vector. This is consistent with other studies describing lower overall anti-PreS2 antibody titers in comparison to anti-PreS1 antibody titers after immunization with DNA vectors encoding the L protein [24] or with a PreS1-, PreS2- and S-containing protein vaccine [27]. When we immunized mice with a mixture of the two DNA vectors encoding the S and the L protein, we did not observe an immunological benefit over immunization with the individual vectors. We therefore discarded the mixture as potential vaccine candidate and proceeded with individual, SAINT-18-formulated MIDGE-Th1 vectors expressing either S or L protein as vaccine candidates. To predict efficacy of these vaccine candidates in humans, we evaluated their immunogenicity in a suitable large animal model and compared them to the protein vaccine Engerix-B. The pig model is considered appropriate as it allows for comparison of vaccines at full human doses. Considering that intradermal administration is the intended clinical route of our DNA vaccine candidates, the morphological similarity of porcine to human skin [28] is relevant. In addition, pigs have been reported to develop immune responses to HBsAg [29], [30], [31]. Delivery of naked HBsAg-encoding DNA vectors by needle and syringe has been reported to induce only weak immune responses in contrast to delivery by electroporation, emphasizing the need for a delivery system to enhance the efficacy of DNA vaccination. Based on our previous studies in mice [20] we hypothesized that formulation with SAINT-18 is an efficient delivery method for MIDGE-Th1 vectors in pigs as well.

Indeed, both vaccine candidates generated S protein-specific antibody levels in pigs that would be considered protective in humans. A single immunization with S protein-encoding vectors achieved 100% seroconversion at all tested DNA doses. In contrast, three immunizations with L protein-encoding vectors at high dose induced only 80% seroconversion at ∼1000-fold lower levels. The effect of low antigen secretion from L protein-encoding vectors on the induction of S protein-specific antibodies was obviously more pronounced in pigs than in mice. The lower immunogenicity of L protein-encoding vectors was also reflected by the weak cellular immune response detected in pigs. Only low amounts of S-specific and no PreS1- and PreS2-specific IFN-γ-secreting PBMC were detected. In mice, strong S-specific [32], [33] and negligible PreS1/PreS2-specific [33] cellular responses have been described after immunization with DNA vaccines expressing non-secreted L proteins. The comparably high DNA doses applied in those studies (15 µg [33] or 100 µg DNA [32] for a ∼20 g mouse) as opposed to 333 µg DNA for a ∼20 kg pig used in our study might explain the different level of S-specific cellular immune responses.

Comparing the immunogenicity of S protein-encoding vectors to Engerix B, we observed a similar level of S protein-specific antibodies and comparable cellular immune responses. Only few side-by-side studies measuring the efficacy of DNA vaccines in comparison to licensed protein vaccines have been performed so far, especially not in relevant large animal models. Babiuk et al. showed 100% seroconversion after two immunizations of pigs with 1000 µg HBsAg-encoding plasmid delivered by electroporation [29] and the same seroconversion rate was postulated for two immunizations with Engerix-B [30]. In chimpanzees, a high dose of plasmid DNA (2 mg) encoding for PreS2 and S protein induced the same level of antibodies as a commercial protein subunit vaccine [34]. In comparison, our study showed that formulation with SAINT-18 is an effective delivery method for DNA vaccines, notably MIDGE-Th1 vectors, since a DNA dose as low as 83 µg was effective in pigs.

For mice, it is established that immunization with DNA and aluminum-hydroxide-adjuvanted protein vaccines against HBV elicits a Th-type 1 (Th1) and Th2 type biased immune response, respectively [35], [36]. In our pig study, we also observed that Engerix B induced high amounts of IgG1 antibodies whereas MIDGE-Th1 vectors induced more IgG2 antibodies. Though the function of Ig isotypes in pigs has not been as extensively described, Th2 cytokines have been shown to increase the IgG1:IgG2 ratio [37], indicating that Ig isotype production in pigs may follow the Th1/Th2 paradigm suggested for mice [38]. However, the apparent difference in the type of humoral immune response was not reflected by the cellular immune responses since the S protein-encoding vector and Engerix-B induced the same level of S protein-specific IFN-γ-secreting PBMC.

The PreS1-specific antibody titers detected in some animals immunized with S protein-encoding vectors, though at a low level, was unexpected. These antibodies might be cross-reactive with low specificity for the PreS1 protein, although sequence alignments revealed no similarity between the PreS1 and S protein amino acid sequence. In this context it is puzzling that the S protein-specific antibodies induced by Engerix-B were not cross-reactive. S protein expressed from DNA in immunized animals differs from recombinant yeast-derived S protein (e.g. folding, glycosylation) and antigen presentation after DNA and protein immunization might deviate as well which potentially influences the development of cross-reactive antibodies.

In humans, antibody levels wane over time after vaccination with recombinant HBsAg protein vaccines [39]. Accordingly, we have observed a decline in antibody levels within 6 months after the third immunization of pigs. Though the low number of animals allows no definitive conclusion, the decline seemed less pronounced in pigs immunized with S protein-encoding MIDGE-Th1 vectors compared to pigs immunized with Engerix-B. Prolonged presence of the MIDGE-Th1 vector in skin and draining lymph nodes [23] resulting in prolonged antigen expression and immune stimulation may account for this effect. Another possible explanation is that the DNA vaccine induced more long-lived antibody-secreting plasma cells [40] maintaining specific antibody levels in the absence of antigen.

From our studies in mice and pigs we conclude that the SAINT-18-formulated MIDGE-Th1 vector encoding the S protein of HBsAg is the most promising vaccine candidate at present. Although immunogenicity in humans remains to be determined, our data indicate that the generation of total S-specific antibodies, the hallmark for licensing of HBV vaccines, can approach that of conventional vaccines after immunization with this vaccine candidate. Although the L protein-encoding vector elicited high PreS1-specific antibody titers in pigs, the level of S-specific antibodies does not suggest that this vaccine candidate can confer protection in humans. Thus, further optimization of the L protein antigen sequence, e.g. allowing for efficient secretion of the L protein [36], would be required prior to clinical testing.

Taken together, we established efficacy of our SAINT-18-formulated MIDGE-Th1 DNA vaccine for prophylaxis of HBV infection in a large animal model. With this relevant proof of concept, the application of SAINT-18 formulated MIDGE-Th1 vectors can be expanded to the development of other prophylactic vaccines with an antibody-based mode of protection against a broad range of pathogens.

## Supporting Information

Table S1 **Statistical analysis for total S protein-specific antibodies in pigs (** **Figure 4A** **).** Other days and group comparisons were not significant.
(DOCX)

Table S2 **Statistical analysis for PreS1-specific IgG in pigs (** **Figure 4B** **).** Other days and group comparisons were not significant.
(DOCX)

Table S3 **Statistical analysis for S protein-specific IgG1 in pigs (** **Figure 4C** **).** Other days and group comparisons were not significant.
(DOCX)

Table S4 **Statistical analysis for S protein-specific IgG2 in pigs (** **Figure 4D** **).** Other days and group comparisons were not significant.
(DOCX)

Table S5 **Selection of pigs for extended study based on total S protein-specific antibody levels on day 71.** Other days and group comparisons were not significant.
(DOCX)

## References

[1] WHO (2009) Hepatitis B vaccines. Wkly Epidemiol Rec 40: 20.

[2] Prange R (2012) Host factors involved in hepatitis B virus maturation, assembly, and egress. Medical microbiology and immunology 201: 449–461. 22965171 10.1007/s00430-012-0267-9

[3] Patient R, Hourioux C, Sizaret PY, Trassard S, Sureau C, et al (2007) Hepatitis B virus subviral envelope particle morphogenesis and intracellular trafficking. Journal of virology 81: 3842–3851. 17267490 10.1128/JVI.02741-06 PMC1866106

[4] Zanetti AR, Van Damme P, Shouval D (2008) The global impact of vaccination against hepatitis B: a historical overview. Vaccine 26: 6266–6273. 18848855 10.1016/j.vaccine.2008.09.056

[5] Kao JH, Chen DS (2002) Global control of hepatitis B virus infection. The Lancet infectious diseases 2: 395–403. 12127351 10.1016/s1473-3099(02)00315-8

[6] Cooper C, Mackie D (2011) Hepatitis B surface antigen-1018 ISS adjuvant-containing vaccine: a review of HEPLISAV safety and efficacy. Expert review of vaccines 10: 417–427. 21506639 10.1586/erv.10.162

[7] Podda A, Del Giudice G (2003) MF59-adjuvanted vaccines: increased immunogenicity with an optimal safety profile. Expert review of vaccines 2: 197–203. 12899571 10.1586/14760584.2.2.197

[8] Tong NK, Beran J, Kee SA, Miguel JL, Sanchez C, et al (2005) Immunogenicity and safety of an adjuvanted hepatitis B vaccine in pre-hemodialysis and hemodialysis patients. Kidney international 68: 2298–2303. 16221232 10.1111/j.1523-1755.2005.00689.x

[9] Tacket CO, Roy MJ, Widera G, Swain WF, Broome S, et al (1999) Phase 1 safety and immune response studies of a DNA vaccine encoding hepatitis B surface antigen delivered by a gene delivery device. Vaccine 17: 2826–2829. 10438052 10.1016/s0264-410x(99)00094-8

[10] Rottinghaus ST, Poland GA, Jacobson RM, Barr LJ, Roy MJ (2003) Hepatitis B DNA vaccine induces protective antibody responses in human non-responders to conventional vaccination. Vaccine 21: 4604–4608. 14575774 10.1016/s0264-410x(03)00447-x

[11] Raz R, Koren R, Bass D (2001) Safety and immunogenicity of a new mammalian cell-derived recombinant hepatitis B vaccine containing Pre-S1 and Pre-S2 antigens in adults. Isr Med Assoc J 3: 328–332. 11411195

[12] Rendi-Wagner P, Shouval D, Genton B, Lurie Y, Rumke H, et al (2006) Comparative immunogenicity of a PreS/S hepatitis B vaccine in non- and low responders to conventional vaccine. Vaccine 24: 2781–2789. 16455169 10.1016/j.vaccine.2006.01.007

[13] Shapira MY, Zeira E, Adler R, Shouval D (2001) Rapid seroprotection against hepatitis B following the first dose of a Pre-S1/Pre-S2/S vaccine. Journal of hepatology 34: 123–127. 11211888 10.1016/s0168-8278(00)00082-9

[14] Neurath AR, Kent SB, Adamowicz P, Riottot MM, Price P, et al (1987) Immunological cross-reactivity between preS2 sequences of the hepatitis B virus envelope proteins corresponding to serological subtypes adw2 and ayw. Mol Immunol 24: 561–568. 3657796 10.1016/0161-5890(87)90036-8

[15] Neurath AR, Kent SB, Parker K, Prince AM, Strick N, et al (1986) Antibodies to a synthetic peptide from the preS 120–145 region of the hepatitis B virus envelope are virus neutralizing. Vaccine 4: 35–37. 2421497 10.1016/s0264-410x(86)80001-9

[16] Moreno S, Lopez-Fuertes L, Vila-Coro AJ, Sack F, Smith CA, et al (2004) DNA immunisation with minimalistic expression constructs. Vaccine 22: 1709–1716. 15068854 10.1016/j.vaccine.2003.09.051

[17] Schirmbeck R, Konig-Merediz SA, Riedl P, Kwissa M, Sack F, et al (2001) Priming of immune responses to hepatitis B surface antigen with minimal DNA expression constructs modified with a nuclear localization signal peptide. Journal of molecular medicine 79: 343–350. 11485031 10.1007/s001090100227

[18] Zheng C, Juhls C, Oswald D, Sack F, Westfehling I, et al (2006) Effect of different nuclear localization sequences on the immune responses induced by a MIDGE vector encoding bovine herpesvirus-1 glycoprotein D. Vaccine. 24: 4625–4629. 10.1016/j.vaccine.2005.08.046 16154243

[19] van der Woude I, Wagenaar A, Meekel AA, ter Beest MB, Ruiters MH, et al (1997) Novel pyridinium surfactants for efficient, nontoxic in vitro gene delivery. Proceedings of the National Academy of Sciences of the United States of America 94: 1160–1165. 9037023 10.1073/pnas.94.4.1160 PMC19761

[20] Endmann A, Baden M, Weisermann E, Kapp K, Schroff M, et al (2010) Immune response induced by a linear DNA vector: influence of dose, formulation and route of injection. Vaccine 28: 3642–3649. 20362204 10.1016/j.vaccine.2010.03.034

[21] Panjaworayan N, Roessner SK, Firth AE, Brown CM (2007) HBVRegDB: annotation, comparison, detection and visualization of regulatory elements in hepatitis B virus sequences. Virology journal 4: 136. 18086305 10.1186/1743-422X-4-136 PMC2235840

[22] Bruss V, Vieluf K (1995) Functions of the internal pre-S domain of the large surface protein in hepatitis B virus particle morphogenesis. Journal of virology 69: 6652–6657. 7474074 10.1128/jvi.69.11.6652-6657.1995 PMC189574

[23] Endmann A, Oswald D, Riede O, Talman EG, Vos RE, et al. (2014) Combination of MIDGE-Th1 DNA vaccines with the cationic lipid SAINT-18: Studies on formulation, biodistribution and vector clearance. Vaccine: In press http://dx.doi.org/10.1016/j.vaccine.2014.1003.1048. 10.1016/j.vaccine.2014.03.048 24681271

[24] Prange R, Werr M (1999) DNA-mediated immunization to hepatitis B virus envelope proteins: preS antigen secretion enhances the humoral response. Vaccine 17: 617–623. 10067665 10.1016/s0264-410x(98)00243-6

[25] Michel ML, Davis HL, Schleef M, Mancini M, Tiollais P, et al (1995) DNA-mediated immunization to the hepatitis B surface antigen in mice: aspects of the humoral response mimic hepatitis B viral infection in humans. Proceedings of the National Academy of Sciences of the United States of America 92: 5307–5311. 7777503 10.1073/pnas.92.12.5307 PMC41683

[26] Geissler M, Tokushige K, Chante CC, Zurawski VR Jr, Wands JR (1997) Cellular and humoral immune response to hepatitis B virus structural proteins in mice after DNA-based immunization. Gastroenterology 112: 1307–1320. 9098017 10.1016/s0016-5085(97)70145-8

[27] Jones CD, Page M, Bacon A, Cahill E, Bentley M, et al (1999) T-cell and antibody response characterisation of a new recombinant pre-S1, pre-S2 and SHBs antigen-containing hepatitis B vaccine; demonstration of superior anti-SHBs antibody induction in responder mice. Vaccine 17: 2528–2537. 10418899 10.1016/s0264-410x(99)00061-4

[28] Vardaxis NJ, Brans TA, Boon ME, Kreis RW, Marres LM (1997) Confocal laser scanning microscopy of porcine skin: implications for human wound healing studies. Journal of anatomy 190 (Pt 4): 601–611. 10.1046/j.1469-7580.1997.19040601.x PMC1467644 9183682

[29] Babiuk S, Baca-Estrada ME, Foldvari M, Storms M, Rabussay D, et al (2002) Electroporation improves the efficacy of DNA vaccines in large animals. Vaccine 20: 3399–3408. 12213410 10.1016/s0264-410x(02)00269-4

[30] Babiuk S, Baca-Estrada ME, Foldvari M, Middleton DM, Rabussay D, et al (2004) Increased gene expression and inflammatory cell infiltration caused by electroporation are both important for improving the efficacy of DNA vaccines. Journal of biotechnology 110: 1–10. 15099900 10.1016/j.jbiotec.2004.01.015

[31] Andrianov AK, DeCollibus DP, Gillis HA, Kha HH, Marin A, et al (2009) Poly[di(carboxylatophenoxy)phosphazene] is a potent adjuvant for intradermal immunization. Proceedings of the National Academy of Sciences of the United States of America 106: 18936–18941. 19864632 10.1073/pnas.0908842106 PMC2770009

[32] Shen M, Wang S, Ge G, Xing Y, Ma X, et al (2010) Profiles of B and T cell immune responses elicited by different forms of the hepatitis B virus surface antigen. Vaccine 28: 7288–7296. 20831917 10.1016/j.vaccine.2010.08.081

[33] Obeng-Adjei N, Hutnick NA, Yan J, Chu JS, Myles DJ, et al (2013) DNA vaccine cocktail expressing genotype A and C HBV surface and consensus core antigens generates robust cytotoxic and antibody responses in mice and Rhesus macaques. Cancer gene therapy 20: 652–662. 24310062 10.1038/cgt.2013.65

[34] Davis HL, McCluskie MJ, Gerin JL, Purcell RH (1996) DNA vaccine for hepatitis B: evidence for immunogenicity in chimpanzees and comparison with other vaccines. Proceedings of the National Academy of Sciences of the United States of America 93: 7213–7218. 8692971 10.1073/pnas.93.14.7213 PMC38962

[35] Luxembourg A, Hannaman D, Wills K, Bernard R, Tennant BC, et al (2008) Immunogenicity in mice and rabbits of DNA vaccines expressing woodchuck hepatitis virus antigens. Vaccine 26: 4025–4033. 18556096 10.1016/j.vaccine.2008.05.021

[36] Ge G, Wang S, Han Y, Zhang C, Lu S, et al (2012) Removing N-terminal sequences in pre-S1 domain enhanced antibody and B-cell responses by an HBV large surface antigen DNA vaccine. PloS one 7: e41573. 22844502 10.1371/journal.pone.0041573 PMC3402421

[37] Crawley A, Wilkie BN (2003) Porcine Ig isotypes: function and molecular characteristics. Vaccine 21: 2911–2922. 12798635 10.1016/s0264-410x(03)00142-7

[38] Finkelman FD, Holmes J, Katona IM, Urban JF Jr, Beckmann MP, et al (1990) Lymphokine control of in vivo immunoglobulin isotype selection. Annual review of immunology 8: 303–333. 10.1146/annurev.iy.08.040190.001511 1693082

[39] Gilca V, De Serres G, Boulianne N, Murphy D, De Wals P, et al (2013) Antibody persistence and the effect of a booster dose given 5, 10 or 15 years after vaccinating preadolescents with a recombinant hepatitis B vaccine. Vaccine 31: 448–451. 23206974 10.1016/j.vaccine.2012.11.037

[40] Manz RA, Hauser AE, Hiepe F, Radbruch A (2005) Maintenance of serum antibody levels. Annual review of immunology 23: 367–386. 10.1146/annurev.immunol.23.021704.115723 15771575

