# Supplementary material for: Cationic Lipid-Formulated DNA Vaccine against Hepatitis B Virus: Immunogenicity of MIDGE-Th1 Vectors Encoding Small and Large Surface Antigen in Comparison to a Licensed Protein Vaccine
Source: PLoS One. 2014 Jul 3;9(7):e101715. doi: 10.1371/journal.pone.0101715 (PMC4081723; doi:10.1371/journal.pone.0101715)
Supplement: Table S1 — Statistical analysis for total S protein-specific antibodies in pigs ( Figure 4A ). Other days and group comparisons were not significant. (DOCX) [file pone.0101715.s001.docx]

**Table S1:**

**Statistical analysis for total S protein-specific antibodies in pigs (Figure 4A).**

| **Day** | **Statistical test** | **Groups** | **p-value** |
| --- | --- | --- | --- |
| 29 | Dunnett | high S / Ctrl. | 0.029 |
|  |  | Engerix-B / Ctrl. | 0.0001 |
|  | Tukey | Engerix-B / low S | 0.016 |
|  |  | Engerix‑B / mid S | 0.009 |
|  |  | Engerix-B / high L | 0.0002 |
| 71 | Dunnett | high S / Ctrl. | 0.011 |
|  |  | Engerix-B / Ctrl. | 0.015 |
|  | Tukey | Engerix-B / high L | 0.019 |
|  |  | high S / high L | 0.012 |

Other days and group comparisons were not significant.
