# Supplementary material for: Cationic Lipid-Formulated DNA Vaccine against Hepatitis B Virus: Immunogenicity of MIDGE-Th1 Vectors Encoding Small and Large Surface Antigen in Comparison to a Licensed Protein Vaccine
Source: PLoS One. 2014 Jul 3;9(7):e101715. doi: 10.1371/journal.pone.0101715 (PMC4081723; doi:10.1371/journal.pone.0101715)
Supplement: Table S2 — Statistical analysis for PreS1-specific IgG in pigs ( Figure 4B ). Other days and group comparisons were not significant. (DOCX) [file pone.0101715.s002.docx]

**Table S2:**

**Statistical analysis for PreS1-specific IgG in pigs (Figure 4B).**

| **Day** | **Statistical test** | **Groups** | **p-value** |
| --- | --- | --- | --- |
| 43 | Dunnett | high L / Ctrl. | 0.021 |
|  | Tukey | high L / low S | 0.024 |
|  |  | high L / mid S | 0.045 |
|  |  | high L / high S | 0.045 |
|  |  | high L / Engerix-B | 0.035 |
| 57 | Dunnett | high L / Ctrl. | 0.01 |
|  | Tukey | high L / low S | 0.011 |
|  |  | high L / mid S | 0.036 |
|  |  | high L / high S | 0.039 |
|  |  | high L / Engerix-B | 0.017 |
| 71 | Dunnett | high L / Ctrl. | 0.00002 |
|  | Tukey | high L / low S | 0.00002 |
|  |  | high L / mid S | 0.00007 |
|  |  | high L / high S | 0.00006 |
|  |  | high L / Engerix-B | 0.00004 |

Other days and group comparisons were not significant.
