# Supplementary material for: Cationic Lipid-Formulated DNA Vaccine against Hepatitis B Virus: Immunogenicity of MIDGE-Th1 Vectors Encoding Small and Large Surface Antigen in Comparison to a Licensed Protein Vaccine
Source: PLoS One. 2014 Jul 3;9(7):e101715. doi: 10.1371/journal.pone.0101715 (PMC4081723; doi:10.1371/journal.pone.0101715)
Supplement: Table S3 — Statistical analysis for S protein-specific IgG1 in pigs ( Figure 4C ). Other days and group comparisons were not significant. (DOCX) [file pone.0101715.s003.docx]

**Table S3:**

**Statistical analysis for S protein-specific IgG1 in pigs (Figure 4C).**

| **Day** | **Statistical test** | **Groups** | **p-value** |
| --- | --- | --- | --- |
| 15 | Dunnett | Engerix-B / Ctrl. | 0.018 |
|  | Tukey | Engerix-B / high L | 0.023 |
| 29 | Dunnett | Engerix-B / Ctrl. | 0.002 |
|  | Tukey | Engerix-B / low S | 0.024 |
|  |  | Engerix‑B / mid S | 0.009 |
|  |  | Engerix‑B / high S | 0.038 |
|  |  | Engerix-B / high L | 0.002 |
| 43 | Dunnett | Engerix-B / Ctrl. | 0.025 |
|  | Tukey | Engerix-B / high L | 0.032 |
| 57 | Dunnett | Engerix-B / Ctrl. | 0.016 |
|  | Tukey | Engerix-B / high L | 0.02 |
| 71 | Dunnett | Engerix-B / Ctrl. | 0.00002 |
|  | Tukey | Engerix-B / low S | 0.002 |
|  |  | Engerix‑B / mid S | 0.0002 |
|  |  | Engerix‑B / high S | 0.001 |
|  |  | Engerix-B / high L | 0.00003 |

Other days and group comparisons were not significant.
