# Supplementary material for: Cationic Lipid-Formulated DNA Vaccine against Hepatitis B Virus: Immunogenicity of MIDGE-Th1 Vectors Encoding Small and Large Surface Antigen in Comparison to a Licensed Protein Vaccine
Source: PLoS One. 2014 Jul 3;9(7):e101715. doi: 10.1371/journal.pone.0101715 (PMC4081723; doi:10.1371/journal.pone.0101715)
Supplement: Table S4 — Statistical analysis for S protein-specific IgG2 in pigs ( Figure 4D ). Other days and group comparisons were not significant. (DOCX) [file pone.0101715.s004.docx]

**Table S4:**

**Statistical analysis for S protein-specific IgG2 in pigs (Figure 4D).**

| **Day** | **Statistical test** | **Groups** | **p-value** |
| --- | --- | --- | --- |
| 15 | Dunnett | high S / Ctrl. | 0.024 |
|  | Tukey | high S / Engerix‑B | 0.05 |
|  |  | high S / high L | 0.03 |
| 43 | Dunnett | low S / Ctrl. | 0.01 |
|  | Tukey | low S / high L | 0.01 |
| 57 | Dunnett | low S / Ctrl. | 0.002 |
|  |  | high S / Ctrl. | 0.035 |
|  | Tukey | low S / Engerix-B | 0.017 |
|  |  | low S / high L | 0.002 |
|  |  | high S / high L | 0.04 |
| 71 | Dunnett | high S / Ctrl. | 0.006 |
|  | Tukey | high S / Engerix‑B | 0.024 |
|  |  | high S / high L | 0.007 |
|  |  | high S / mid S | 0.026 |

Other days and group comparisons were not significant.
