# Supplementary material for: Cationic Lipid-Formulated DNA Vaccine against Hepatitis B Virus: Immunogenicity of MIDGE-Th1 Vectors Encoding Small and Large Surface Antigen in Comparison to a Licensed Protein Vaccine
Source: PLoS One. 2014 Jul 3;9(7):e101715. doi: 10.1371/journal.pone.0101715 (PMC4081723; doi:10.1371/journal.pone.0101715)
Supplement: Table S5 — Selection of pigs for extended study based on total S protein-specific antibody levels on day 71. Other days and group comparisons were not significant. (DOCX) [file pone.0101715.s005.docx]

**Table S5:**

**Selection of pigs for extended study based on total S protein-specific antibody levels on day 71.**

| **Group name** | **Animal No.** | **Total anti-S (mIU/ml)** |
| --- | --- | --- |
| low S | **7** | **1.84x10^4^** |
|  | 8 | 5.37x10^3^ |
|  | **9** | **4.22x10^4^** |
|  | 10 | 4.18x10^4^ |
|  | 11 | 2.80x10^4^ |
|  | Mean | 2.72x10^4^ |
| high S | 17 | 3.77x10^4^ |
|  | **18** | **1.24x10^5^** |
|  | 19 | 3.08x10^4^ |
|  | **20** | **3.17x10^4^** |
|  | 21 | 4.25x10^4^ |
|  | Mean | 5.33x10^4^ |
| Engerix-B | **32** | **8.27x10^4^** |
|  | 33 | 3.33x10^4^ |
|  | 34 | 4.40x10^4^ |
|  | **35** | **5.33x10^4^** |
|  | Mean | 5.33x10^4^ |

Pigs selected for further monitoring are highlighted in bold. One animal with the highest response was selected of each group as well as one animal exhibiting a response in the range of the mean antibody level of the group.
